# Parameter-resolved AC electrical stimulation links electrical system characteristics to early transcriptional responses in human stem cells

**DOI:** 10.3389/fbioe.2026.1897534

**Published:** 2026-08-25

**Authors:** Laura Lembcke, Henning Bathel, Bernhard Frerich, Michael Dau, Ursula van Rienen, Peer W. Kämmerer, Nadja Engel

**Affiliations:** 1 Department of Oral and Maxillofacial Surgery, Facial Plastic Surgery, Rostock University Medical Center, Rostock, Germany; 2 Department of Oral and Maxillofacial Surgery, Facial Plastic Surgery, University Medical Center Mainz, Mainz, Germany; 3 Institute of General Electrical Engineering, University of Rostock, Rostock, Germany; 4 Department of Life, Light and Matter, University of Rostock, Rostock, Germany; 5 Department of Ageing of Individuals and Society, Interdisciplinary Faculty, University of Rostock, Rostock, Germany; 6 Oscar Langendorff Institute of Physiology, University of Rostock, Rostock, Germany

**Keywords:** alternating current, electrical stimulation, frequency-dependent effects, mesenchymal stem cells, osteogenesis-associated gene expression, sinusoidal AC stimulation

## Abstract

Alternating-current electrical stimulation (AC-ES) may modulate stem-cell responses, but nominal stimulation settings do not define the electric-field exposure at the cell layer, limiting cross-study comparison. Sinusoidal AC-ES was applied to human adipose-derived stem cells (hASC) and human bone marrow-derived mesenchymal stem cells (hMSC-BM) using a validated *in vitro* framework that combines continuous electrical monitoring with experimentally parameterized, electrode–electrolyte-interface-informed finite-element simulations. The study was designed to relate physically characterized stimulation conditions to early molecular readouts. Electrical measurements revealed frequency-dependent system behavior, while simulations showed amplitude-dependent shifts in cell-layer exposure across defined electric-field ranges. At 20 Hz and 2 Vrms, where cell-layer exposure shifted from predominantly below 5 Vm^-1^ towards the 5–15 Vm^-1^ range, osteogenesis- and stress-associated gene expression changed. Higher amplitudes at 20 Hz reduced selected osteogenesis-associated transcripts in hASC, whereas hMSC-BM showed a comparatively stronger osteogenesis-associated response. Markers related to cell death, survival, and proliferation were selectively regulated, while metabolic activity remained comparable with unstimulated controls. Overall, this workflow identifies biologically compatible, parameter-defined AC-ES conditions and supports the rational selection of stimulation settings for subsequent experiments.

## 1 Introduction

Studies over several decades have demonstrated that electric fields can modulate the behavior of eukaryotic cells, highlighting their therapeutic potential across neural, sensory, musculoskeletal, and stem-cell-based applications (Humayun et al., 1999; Serena et al., 2009; Tamaki et al., 2017; Tandon et al., 2011; Tehovnik, 1996). Clinically, controlled electrical stimulation (ES) is established for excitable tissues, including implantable pacemakers in cardiology (Beck et al., 2010), deep brain stimulation for movement disorders in neurology (Benabid, 2003), and transcutaneous electrical nerve stimulation in pain management (Naka et al., 2013). Beyond excitable tissues, biophysical cues are increasingly recognized as relevant regulators of regeneration and stem-cell fate. In bone tissue engineering, structural, mechanical, and electromagnetic stimuli may complement biomaterial-, cell-, and factor-based strategies by influencing cellular adhesion, migration, proliferation, stress adaptation, and lineage commitment (Hao et al., 2021). Bone is particularly relevant in this context because mechanical loading generates local electrical and electrochemical signals, including piezoelectric effects within the collagen-containing matrix (Shamos et al., 1963). Such endogenous signals are associated with bone adaptation, remodeling, fracture healing, and regeneration (Kraus, 1984; Leppik et al., 2020; Wieland et al., 2015). Recent concepts in electroactive biomaterials further underline the relevance of bioelectric microenvironments for regenerative applications (Hao et al., 2025).

Accordingly, ES has been investigated as a potential biophysical modulator of bone-associated cellular processes. Osteoblasts are sensitive to electric fields, and the mode of electrical coupling is a critical determinant of whether physiological biomineralization is promoted (Wiesmann et al., 2000). In the low-frequency alternating-current (AC) range, voltage- and frequency-dependent effects on preosteoblast gene expression have been reported, with moderate stimulation at a root-mean-square voltage (Vrms) of 0.7 V and 20 Hz inducing early upregulation of osteogenic markers (Sahm et al., 2022). Further studies using human osteoblasts showed that low to moderate voltages of 0.2–1.4 Vrms at 20 Hz enhanced osteogenic gene expression, alkaline phosphatase activity, and collagen type I synthesis, whereas higher voltages exerted inhibitory effects (Sahm et al., 2020). Moreover, alternating electric current at 10 or 40 mA and 10 Hz promoted the differentiation of adult human mesenchymal stem cells towards the osteoblastic lineage, inducing early and late osteogenic markers without activating adipogenic or chondrogenic pathways (Creecy et al., 2013). In human mesenchymal stem cells, exposure to a higher-frequency electric field of 60 kHz and 2 Vm^-1^ enhanced osteogenic marker expression, collagen deposition, and later-stage mineralization (Hronik-Tupaj et al., 2011). Together, these findings support the capacity of ES to influence osteogenesis-associated processes, while also indicating that outcomes depend strongly on the selected stimulation conditions and may involve concurrent stress-associated pathways (Bielfeldt et al., 2022; Love et al., 2017).

Despite substantial progress in developing *in vitro* ES models, reproducibility and cross-study comparison remain major methodological challenges (Budde et al., 2019). Protocols differ substantially with regard to the mode of electrical coupling, current type, waveform, frequency, voltage, or current amplitude, electrode material and geometry, cell source, culture conditions, exposure duration, and biological endpoint (Leppik et al., 2020; Meng et al., 2022; Pettersen et al., 2022). In particular, Pettersen et al. (2022) identified nonuniform protocols and incomplete reporting of stimulation parameters across *in vitro* and *in vivo* studies investigating electrically stimulated osseointegration (Pettersen et al., 2022). Recent work has further emphasized that a stimulation protocol cannot be considered independently of the device in which it is implemented, as nominal voltage, current, frequency, or waveform settings cannot be directly transferred between systems without accounting for their respective physical and electrochemical properties (Kulkarni et al., 2026; Zimmermann et al., 2021). Consequently, although parameter-dependent effects have been reported repeatedly, no consensus currently exists on universally applicable AC stimulation parameters or electric-field ranges for osteogenic *in vitro* experiments. Precise field characterization, transparent reporting of system properties, and standardized stimulation protocols are therefore required to improve reproducibility and mechanistic interpretation (Hlavac et al., 2021; Ryan et al., 2021; Zimmermann et al., 2023).

A major reason for this limited comparability is that nominal electrical input settings do not necessarily represent the exposure experienced by adherent cells. In electrode-based stimulation systems, including implant-oriented configurations (Hiemer et al., 2016), the delivered stimulus is defined not only by the applied voltage or current but also by electrode-specific boundary conditions at the electrode–electrolyte interface (EEI) and their coupling to the conductive culture medium (Lembcke et al., 2026). As a result, the effective electric field at the cell layer may differ substantially from the nominal stimulation setting because of spatial inhomogeneity and interface-related effects. Low-frequency stimulation within mechanically relevant frequency ranges has been widely applied in bone-related ES studies because it approximates endogenous electrical signals associated with mechanical loading and has been linked to osteogenic gene regulation (Dauben et al., 2016; Hiemer et al., 2018; Soda et al., 2008). However, this frequency range is also characterised by pronounced EEI contributions, including capacitive double-layer charging and polarization phenomena, which can influence the effective electrical boundary conditions and the resulting field distribution within the culture medium (Franks et al., 2005; Richardot and McAdams, 2002; Zimmermann et al., 2023). Therefore, field strength cannot be adequately represented by a single scalar value in spatially inhomogeneous configurations. Instead, experimentally informed and EEI-aware finite-element (FE) modeling is required to estimate local exposure conditions and relate physical stimulation parameters to cellular readouts (Budde et al., 2019; Zimmermann et al., 2022; 2023).

In our previous work, we established and validated a measurement-based *in vitro* framework for sinusoidal low-frequency AC stimulation of adherent cells, using human adipose-derived stem cells (hASC) as a biological model system (Lembcke et al., 2026). During stimulation, the applied input voltage and electrode-specific output voltages were repeatedly recorded under cell-culture conditions. These signals were used to derive electrode-specific current, impedance magnitude, and phase shift, thereby characterizing the time-resolved electrical properties of the EEI. The resulting measurements, including the effective voltage across the electrode–medium system, were subsequently incorporated as boundary conditions into FE simulations. This enabled estimation of the spatial electric-field distribution at the cell layer based on experimentally monitored system characteristics and EEI-informed electrical conditions rather than on nominal stimulation settings alone. Using this approach, the previous study identified a system-specific, biologically compatible operating window under the investigated 20 Hz conditions (Lembcke et al., 2026).

Building on this framework, the present study was designed to support future ES experiments by narrowing the relevant parameter space and relating physically characterized AC stimulation conditions to early molecular response patterns. It was not intended as a comprehensive endpoint analysis of osteogenic differentiation. The hASC and human bone marrow-derived mesenchymal stem cells (hMSC-BM) were continuously exposed to sinusoidal AC stimulation for 3 days at 20 Hz and 1 kHz across multiple voltage amplitudes. The highest tested continuous amplitudes, 3 Vrms at 20 Hz and 1.2 Vrms at 1 kHz, were included to probe the upper part of the parameter space within this specific experimental system and to identify conditions associated with preserved cellular compatibility or emerging stress-associated changes. The resulting patterns are therefore specific to the applied electrode configuration, culture conditions, cell populations, EEI characteristics, and continuous three-day exposure protocol and should not be interpreted as universally transferable thresholds. Although low-frequency sinusoidal stimulation at 20 Hz has informed previous *in vitro*, *in vivo*, and *in silico* approaches, largely based on empirical observations of bone-regenerative field ranges, each device, cell population, and application context requires separate validation (Kraus, 1984; Raben et al., 2019; 2024). Previous continuous 20-Hz hASC experiments further illustrate that cellular outcomes may change with exposure duration (Kämmerer et al., 2020). Parameter-specific transcriptional regulation was assessed using a targeted gene panel covering osteogenesis-associated markers (*ALPL*, *COL1A1*, *SPARC*), cellular stress responses (*HSPB1*, *HSPA1A*), regulated cell death- and survival-associated pathways (*CASP3*, *MLKL*, *GPX4*), and cell-cycle control/proliferation (*CCNB1*, *CDKN1A*, *MKI67*) (Dauben et al., 2016; Forcina and Dixon, 2019; Hiemer et al., 2016; Hronik-Tupaj et al., 2011; Rosado et al., 2006; Sahm et al., 2020; Shende et al., 2019). By integrating viability assessment and early gene-expression profiling with experimentally measured electrical system properties and cell-layer-resolved electric-field exposure, this study aims to identify biologically compatible, parameter-defined AC stimulation conditions that can guide the selection of settings for subsequent experiments.

## 2 Materials and methods

### 2.1 Isolation and culture of human stem cells

Human adipose-derived stem cells (hASC) were isolated from lipoaspirates obtained during elective liposuction procedures at the University Medical Center Rostock, Germany, following approval by the local ethics committee (ethics approval no. A 2025-0039). Isolation was performed according to the established protocols described by (Zuk et al. 2001; Zuk et al. 2002). Briefly, lipoaspirates were enzymatically digested with collagenase, followed by centrifugation and filtration. The resulting stromal vascular fraction was resuspended in culture medium and expanded under standard cell culture conditions. Cell characterization was performed in accordance with the minimal criteria defined by the International Society for Cellular Therapy (Dominici et al., 2006), and the hASC population used in this study has been characterized and reported in detail previously (Lembcke et al., 2026). To additionally verify the osteogenic differentiation capacity of both cell populations, hASC and hMSC-BM were cultured for 21 days in osteogenic differentiation medium (StemMACS™ OsteoDiff Medium, Miltenyi Biotec, Bergisch Gladbach, Germany) and stained with Alizarin Red S (Merck KGaA, Darmstadt, Germany) according to the manufacturer’s protocols. Representative micrographs of differentiated cultures and corresponding growth-medium controls are shown in Supplementary Figure 1.

The culture medium for hASC consisted of 44.5% Gibco™ Iscove’s Modified Dulbecco’s Medium (IMDM) with GlutaMAX™ supplement (Thermo Fisher Scientific, Waltham, USA), 44.5% Gibco™ Ham’s F-12 Nutrient Mix with GlutaMAX™ supplement (Thermo Fisher Scientific), 10% Newborn Calf Serum (HyClone™, Cytiva, Marlborough, USA), and 1% Gibco™ penicillin-streptomycin (5000 U/mL; Thermo Fisher Scientific). hMSC-BM were obtained commercially from PromoCell GmbH (Heidelberg, Germany) and cultured according to the manufacturer’s instructions in Mesenchymal Stem Cell Growth Medium 2 (PromoCell GmbH), supplemented with 1% penicillin-streptomycin (5000 U/mL; Thermo Fisher Scientific). For routine passaging and experimental preparation, adherent stem cells were detached using Gibco™ Trypsin-EDTA (0.05%; Thermo Fisher Scientific) according to the manufacturer’s recommendations.

### 2.2 Electrical stimulation protocol

ES experiments were conducted using a custom-developed *in vitro* system applying sinusoidal AC, as described in detail previously (Lembcke et al., 2026). Cells were continuously stimulated for 3 days. At 20 Hz, root mean square voltages of 1, 2, and 3 Vrms were applied, whereas at 1 kHz, voltages of 0.6 and 1.2 Vrms were used. The applied voltage levels were defined based on the input voltage measured with an oscilloscope rather than the nominal function generator settings. Consequently, throughout this study, the term “applied voltage” refers exclusively to the experimentally verified voltage delivered to the stimulation system, as determined by oscilloscope-based measurements.

### 2.3 Execution of electrical stimulation and numerical electric field simulation

ES experiments were performed using Ti6Al4V electrodes in an implant-oriented stimulation configuration, as established in previous bone- and cartilage-related *in vitro* ES studies (Dauben et al., 2016; Hiemer et al., 2016; 2018). Electrodes were arranged in a 6-well format and operated in the low configuration with an electrode-to-well-bottom distance of 1 mm (Hiemer et al., 2018; Zimmermann et al., 2023). Ti6Al4V was selected because of its clinical relevance as a biomedical implant material, including its mechanical stability, corrosion resistance, and biocompatibility, although its electrochemical performance is less favorable than that of classical bioelectrode materials such as platinum (Khorasani et al., 2015; Kulkarni et al., 2026; Mobini et al., 2022; Niinomi, 1998).

The ES system enabled automated oscilloscope-based recording of the applied input voltage and electrode-specific output voltages at predefined intervals during stimulation, as described in detail previously (Lembcke et al., 2026). During stimulation, the applied voltage (V_high_) and the voltage drop across each electrode, measured via a 10 Ω shunt resistor (V_low_), were recorded once per hour. The effective voltage across the electrode-medium system (V_total_) was calculated as V_total_ = V_high_ − V_low_. Based on these recordings, electrode-specific current, impedance magnitude, and phase shift were calculated and evaluated over the complete three-day stimulation period separately for hASC and hMSC-BM. Electrical stability and frequency-dependent impedance behavior were analyzed for each voltage condition. All processed output data are provided in Supplementary Material 2.

Numerical electric field simulations were performed for each ES condition using the FE method implemented in Netgen and NGSolve (Schöberl, 1997; 2014), following the modeling framework described previously (Lembcke et al., 2026; Zimmermann et al., 2023). A symmetric impedance distribution was assumed. Simulations were parameterized using experimentally determined impedance values, V_total_, and the electrical conductivity of the respective culture media at 37 °C. The electrical conductivity of the culture media was determined by impedance spectroscopy using a NEISYS impedance analyzer (Novocontrol Technologies GmbH & Co. KG, Montabaur, Germany) equipped with a Novocontrol BDS 1309 liquid sample cell. Measurements were performed over a frequency range from 5 kHz to 5 mHz. Conductivity was derived from the frequency-independent bulk resistance plateau in the kHz range, where electrode polarization effects are negligible. The resulting conductivity values were 2.0 S/m for hASC medium and 2.1 S/m for hMSC-BM medium and were treated as constant parameters in the simulations.

For quantitative evaluation of cellular exposure, the electric field magnitude was analyzed at a biologically relevant height of 4 µm above the well bottom, corresponding to the adherent cell layer. Adherent mesenchymal stem cells exhibit a flattened morphology with a cell height in the lower micrometer range (Docheva et al., 2008). Validation simulations showed no relevant differences in electric field magnitude at 2 μm, 4 μm, and 6 µm above the culture surface; therefore, 4 µm was selected as a representative cell-level reference position (Lembcke et al., 2026). A horizontal slice was extracted at this height, and the spatial distribution of the electric field magnitude was segmented into predefined field strength ranges (<5, 5–15, 15–25, 25–35, and >35 V/m).

For visualization, the ES experiment and electrode whose electric field values at the central evaluation position were closest to the mean across all ES experiments were selected. In addition, the simulation time point closest to the temporal mean of the three-day stimulation period was chosen. Representative electric field distributions at 4 µm height were visualized using ParaView (v6.0.1; Kitware Inc., Clifton Park, USA). All numerical simulation data are provided in Supplementary Material 1 and are publicly available via Zenodo (https://doi.org/10.5281/zenodo.18466464).

### 2.4 Preparation of cells and electrodes for electrical stimulation

Ti6Al4V electrodes were prepared as described previously (Lembcke et al., 2026). Briefly, electrodes were cleaned by sequential treatment with 70% ethanol (PKH GmbH, Halle, Germany) and 5% acetic acid (University Medicine Rostock Pharmacy, Rostock, Germany), rinsed with distilled water after each cleaning step, and autoclaved at 121 °C for 20 min at 2.1 bar prior to use. Electrodes were placed into the wells immediately before stimulation and connected to the ES system using crocodile clamps. The stimulation hardware and electrode-containing culture plates were pre-equilibrated under incubator conditions before the start of ES. Unstimulated controls were prepared identically but remained electrically unconnected.

The hASC and hMSC-BM cells were used between passages 3 and 7. For viability and gene expression analyses, cells were seeded at a density of 0.6 × 10^5^ cells per well in 6-well culture plates. After a 24 h adhesion period, cells were washed once with 1× phosphate-buffered saline (Merck KGaA, Darmstadt, Germany) and supplied with 7.5 mL of fresh cell-type-specific culture medium. ES was initiated after approximately 45 min of incubator equilibration.

### 2.5 Cell viability assay

Metabolic activity was assessed after 3 days of ES using an XTT assay (Merck KGaA) according to the manufacturer’s instructions. The XTT reagent, supplemented with an electron-coupling solution, was added directly to the culture medium and incubated for 1 h at 37 °C. Absorbance was measured photometrically at 492 nm using 620 nm as a reference wavelength with an Absorbance 96 plate reader (Byonoy GmbH, Hamburg, Germany). Values were normalized to unstimulated controls, which were set to 100%, and are presented as relative percentages.

### 2.6 Gene expression analysis

After 3 days of ES, total RNA was isolated using a commercial kit (innuPREP RNA Mini Kit 2.0, IST Innuscreen, Berlin, Germany) according to the manufacturer’s instructions. RNA concentration and integrity were determined using the Qubit™ RNA Broad Range (BR) Assay Kit and Qubit™ RNA IQ Assay Kit (Thermo Fisher Scientific). Complementary DNA (cDNA) was synthesized from 300 ng total RNA per sample using the RevertAid First Strand cDNA Synthesis Kit (Thermo Fisher Scientific) in a final reaction volume of 20 µL. The present study focuses on early transcriptional responses to AC-ES within defined short-term stimulation windows. Accordingly, gene expression analyses were performed to capture initial molecular responses related to osteogenesis-associated gene expression, cellular stress regulation, survival-associated pathways, and cell cycle control. Quantitative real-time PCR was performed using a qTOWER^3^ system (Analytik Jena, Jena, Germany) with the Luna® Universal qPCR Master Mix (New England Biolabs, Ipswich, USA). Based on prior validation of housekeeping gene stability under ES conditions, 18S rRNA was used for normalization.

Primers for *HSPB1*, *HSPA1A*, *CASP3*, *MLKL*, *GPX4*, *CCNB1*, *CDKN1A*, and *MKI67* were obtained from OriGene Technologies (Rockville, United States). Primer sequences for *ALPL* and *COL1A1* were adopted from Dauben et al. (2016). Primers for *SPARC* and 18S rRNA were designed using the NCBI Primer-BLAST tool to ensure specificity and optimal amplification parameters. All primer sequences are listed in Table 1, and all primers were synthesized by Thermo Fisher Scientific. Primer efficiency was validated by standard curve analysis using cDNA from native hASC and hMSC-BM cultures. Amplification efficiencies ranged between 90% and 110%, and specificity was confirmed by melt curve analysis. Relative gene expression was calculated using the ΔΔCt method and is presented as log_2_-transformed fold changes (Livak and Schmittgen, 2001; Schmittgen and Livak, 2008).

**TABLE 1:** Overview of primer sequences used for real-time quantitative PCR (RT-qPCR).

HGNC-Symbol/HGNC ID	RefSeq transcript ID/NCBI Gene ID	Forward sequence (5′–3′)	Reverse sequence (5′–3′)	References/derived from
18S rRNA HGNC:10,401	RefSeq NM_022551	GCA​GAA​TCC​ACG​CCA​GTA​CAA​G	GCT​TGT​TGT​CCA​GAC​CAT​TGG​C	-
ALPL HGNC:438	Gene ID 249	CAT​TGT​GAC​CAC​CAC​GAG​AG	CCA​TGA​TCA​CGT​CAA​TGT​CC	Dauben et al. (2016)
COL1A1 HGNC:2197	Gene ID 1277	ACG​AAG​ACA​TCC​CAC​CAA​TC	AGA​TCA​CGT​CAT​CGC​ACA​AC	
SPARC HGNC:11,219	Gene ID 6678	GAT​GGT​GCA​GAG​GAA​ACC​GA	TTT​GCA​AGG​CCC​GAT​GTA​GT	-
HSPB1 HGNC:5246	RefSeq NM_001540	CTG​ACG​GTC​AAG​ACC​AAG​GAT​G	GTG​TAT​TTC​CGC​GTG​AAG​CAC​C	OriGene HP205401
HSPA1A HGNC:5232	RefSeq NM_005345	ACC​TTC​GAC​GTG​TCC​ATC​CTG​A	TCC​TCC​ACG​AAG​TGG​TTC​ACC​A	OriGene HP208499
CASP3 HGNC:1504	RefSeq NM_004346	GGA​AGC​GAA​TCA​ATG​GAC​TCT​GG	GCA​TCG​ACA​TCT​GTA​CCA​GAC​C	OriGene HP207674
MLKL HGNC:26,617	RefSeq NM_152,649	TCA​CAC​TTG​GCA​AGC​GCA​TGG​T	GTA​GCC​TTG​AGT​TAC​CAG​GAA​GT	OriGene HP217870
GPX4 HGNC:4,556	RefSeq NM_002085	ACA​AGA​ACG​GCT​GCG​TGG​TGA​A	CCA​CAC​ACT​TGT​GGA​GCT​AGA	OriGene HP225818
CCNB1 HGNC:1579	RefSeq NM_031966	GAC​CTG​TGT​CAG​GCT​TTC​TCT​G	GGT​ATT​TTG​GTC​TGA​CTG​CTT​GC	OriGene HP215700
CDKN1A HGNC:1784	RefSeq NM_000389	AGG​TGG​ACC​TGG​AGA​CTC​TCA​G	TCC​TCT​TGG​AGA​AGA​TCA​GCC​G	OriGene HP200369
MKI67 HGNC:7,107	RefSeq NM_002417	GAA​AGA​GTG​GCA​ACC​TGC​CTT​C	GCA​CCA​AGT​TTT​ACT​ACA​TCT​GCC	OriGene HP206104

The table lists the official gene symbols and corresponding identifiers (IDs) assigned by the HUGO Gene Nomenclature Committee (HGNC), transcript or gene identifiers from the National Center for Biotechnology Information (NCBI), forward and reverse primer sequences (5′–3′), and the respective reference or source.

### 2.7 Statistical analysis

All ES experiments were performed as technical triplicates using the same biological cell population for each cell type, namely, hASC or hMSC-BM. Thus, the data represent repeated technical measurements within a single biological replicate. For hASC exposed to 3 Vrms at 20 Hz, four technical replicates were included. Data are presented as mean ± standard deviation (SD). Statistical analyses were conducted using GraphPad Prism version 10.4.1 (GraphPad Software, San Diego, USA). Normality of data distribution was assessed using the Shapiro-Wilk test. Cell viability data were analyzed using one-sample t-tests against a normalized reference value of 100 after confirming normality. Gene expression data were analyzed using one-sample t-tests against 0, corresponding to log_2_ (fold change), as well as ordinary one-way analysis of variance (ANOVA) with Tukey’s multiple comparisons test to explore voltage- and frequency-dependent effects.

A p-value < 0.05 was considered statistically significant. In the present study, results from the one-sample t-tests are reported in the main manuscript. The complete statistical evaluation, including results from both statistical approaches and corresponding graphical representations, is provided in Supplementary Material 2. All supplementary datasets supporting this study are available via Zenodo at https://doi.org/10.5281/zenodo.18466464.

## 3 Results

### 3.1 Quantitative analysis of electrical stimulation parameters and numerical field simulation

The effective voltage across the electrode-medium system (V_total_), current, impedance magnitude, and phase shift were evaluated over the complete three-day stimulation period for each ES experiment. Mean values and standard deviations for all stimulation conditions in hASC and hMSC-BM cultures are summarized in Table 2.

**TABLE 2:** Electrical parameters recorded during 3 days of *in vitro* ES with sinusoidal AC at 20 Hz and 1 kHz. Data are shown for applied stimulation amplitudes (V_high_) of 1, 2, and 3 Vrms at 20 Hz and 0.6 and 1.2 Vrms at 1 kHz.

V_high_/Vrms		Freq./Hz	V_total_/Vrms		I/mA		|Z|/Ω		φ/rad	
			mean	SD	mean	SD	mean	SD	mean	SD
hASC	1	20	0.99	0.01	2.2	0.1	458.6	26.4	−0.6	0.1
	2		1.95	0.01	4.9	0.7	409.1	65.2	−0.7	0.1
	3		2.93	0.07	10.8	0.5	270.5	7.5	−0.7	0.0
	0.6	1000	0.56	0.01	4.5	0.6	128.1	20.3	−0.6	0.1
	1.2		1.08	0.02	10.5	1.3	104.0	11.2	−0.5	0.1
hMSC-BM	1	20	1.00	0.01	2.1	0.6	522.5	197.9	−0.7	0.1
	2		1.98	0.04	5.5	0.6	370.5	43.0	−0.8	0.0
	3		2.94	0.05	9.9	0.6	297.7	14.6	−0.7	0.0
	0.6	1000	0.55	0.01	5.5	1.3	104.3	26.6	−0.5	0.2
	1.2		1.09	0.02	11.2	2.0	101.3	21.8	−1.6	2.7

The table summarizes the total effective voltage (V_total_), current (I), impedance magnitude (|Z|), and phase angle (φ) as time-averaged mean ± SD values based on hourly measurements over the complete stimulation period, reported separately for hASC and hMSC-BM.

At 20 Hz, V_total_ closely matched the applied input voltage in both cell culture media, reaching approximately 97%–100% of the set values. Increasing V_total_ was accompanied by a stepwise increase in current and a corresponding decrease in impedance magnitude. Across the tested voltage range, current increased from approximately 2 mA at 1 Vrms to approximately 10–11 mA at 3 Vrms, whereas impedance decreased from approximately 460–520 Ω to approximately 270–300 Ω.

At 1 kHz, Vtotal reached approximately 90%–93% of the applied input voltage. Despite the lower effective voltage, current amplitudes were higher than at low-voltage 20 Hz stimulation and increased from approximately 4.5–5.5 mA at 0.6 Vrms to approximately 10.5–11.2 mA at 1.2 Vrms. Impedance magnitude was substantially lower than at 20 Hz and remained within a comparatively narrow range of approximately 100–130 Ω.

Overall, the electrical characteristics were highly comparable between hASC and hMSC-BM cultures. Stimulation at 1 kHz was associated with lower impedance magnitude and higher current amplitudes than stimulation at 20 Hz at lower voltage amplitudes. Notably, the 20 Hz condition at 3 Vrms generated current amplitudes of 10.8 ± 0.5 mA in hASC and 9.9 ± 0.6 mA in hMSC-BM, which were comparable to those measured at 1 kHz and 1.2 Vrms, with 10.5 ± 1.3 mA and 11.2 ± 2.0 mA, respectively. This indicates that frequency-dependent impedance behavior substantially influenced the delivered current despite comparable or lower effective voltages.

Numerical simulations of the electric field were performed for all ES conditions. The resulting cell-layer electric field exposure was quantified at 4 µm above the well bottom and summarized in Table 3 as area fractions within predefined electric field ranges, reported separately for hASC and hMSC-BM because simulations were parameterized using cell-type-specific medium conductivities.

**TABLE 3:** Spatial distribution of the electric field magnitude at 4 µm above the well bottom for hASC and hMSC-BM during sinusoidal AC-ES.

ES	V_high_/Vrms	Frequency/Hz	Area fraction/%				
			Experiment-specific ranges/Vm^-1^				
			<5	5–15	15–25	25–35	>35
hASC	1	20	88.3	11.7	0.0	0.0	0.0
	2		60.2	33.1	6.7	0.0	0.0
	3		13.2	60.2	14.3	6.0	6.2
	0.6	1000	69.6	25.8	4.6	0.0	0.0
	1.2		20.6	57.2	12.2	5.0	5.0
hMSC-BM	1	20	89.6	10.4	0.0	0.0	0.0
	2		69.0	24.9	6.0	0.1	0.0
	3		19.2	59.5	12.5	5.1	3.8
	0.6	1000	64.2	29.5	6.3	0.0	0.0
	1.2		21.6	57.2	11.9	4.9	4.4

Values represent the percentage surface area, reported as area fraction (%), within experiment-specific electric field ranges (<5, 5–15, 15–25, 25–35, and >35 Vm^-1^) under stimulation at 20 Hz (1, 2, and 3 Vrms) and 1 kHz (0.6 and 1.2 Vrms). Area fractions were derived from FE simulations parameterized with condition-specific electrical properties. Highlighted values indicate the dominant electric field range for each stimulation condition, defined as the range with the maximum area fraction.

Spatial analysis at 4 µm above the well bottom revealed a voltage-dependent redistribution of electric field exposure across defined field strength ranges for both hASC and hMSC-BM. At 20 Hz and 1 Vrms, the vast majority of the culture surface was exposed to field magnitudes below 5 Vm^-1^, corresponding to approximately 88%–90% of the analyzed area. Increasing the amplitude to 2 Vrms reduced the <5 Vm^-1^ fraction and increased the 5–15 Vm^-1^ and 15–25 Vm^-1^ fractions, although <5 Vm^-1^ remained the dominant exposure range. At 3 Vrms, the dominant exposure shifted to the 5–15 Vm^-1^ range, accompanied by substantial contributions in the 15–25 Vm^-1^ range and measurable area fractions above 25 Vm^-1^.

At 1 kHz, a comparable voltage-dependent shift was observed. Stimulation at 0.6 Vrms was primarily associated with field strengths below 5 Vm^-1^, accounting for approximately 64%–70% of the analyzed area. Increasing the amplitude to 1.2 Vrms shifted the dominant exposure to the 5–15 Vm^-1^ range and increased the contribution of higher field bins, including the 15–25 Vm^-1^ and >25 Vm^-1^ ranges. Notably, the spatial exposure profile at 1 kHz and 0.6 Vrms resembled that observed at 20 Hz and 2 Vrms, whereas 1 kHz and 1.2 Vrms produced a distribution comparable to 20 Hz and 3 Vrms, particularly with respect to the dominant 5–15 Vm^-1^ fraction and the emergence of higher field contributions. Across all conditions, spatial distribution patterns were highly similar between hASC and hMSC-BM, indicating comparable cell-layer electric field exposure despite minor differences in medium conductivity.

**FIGURE 1:**
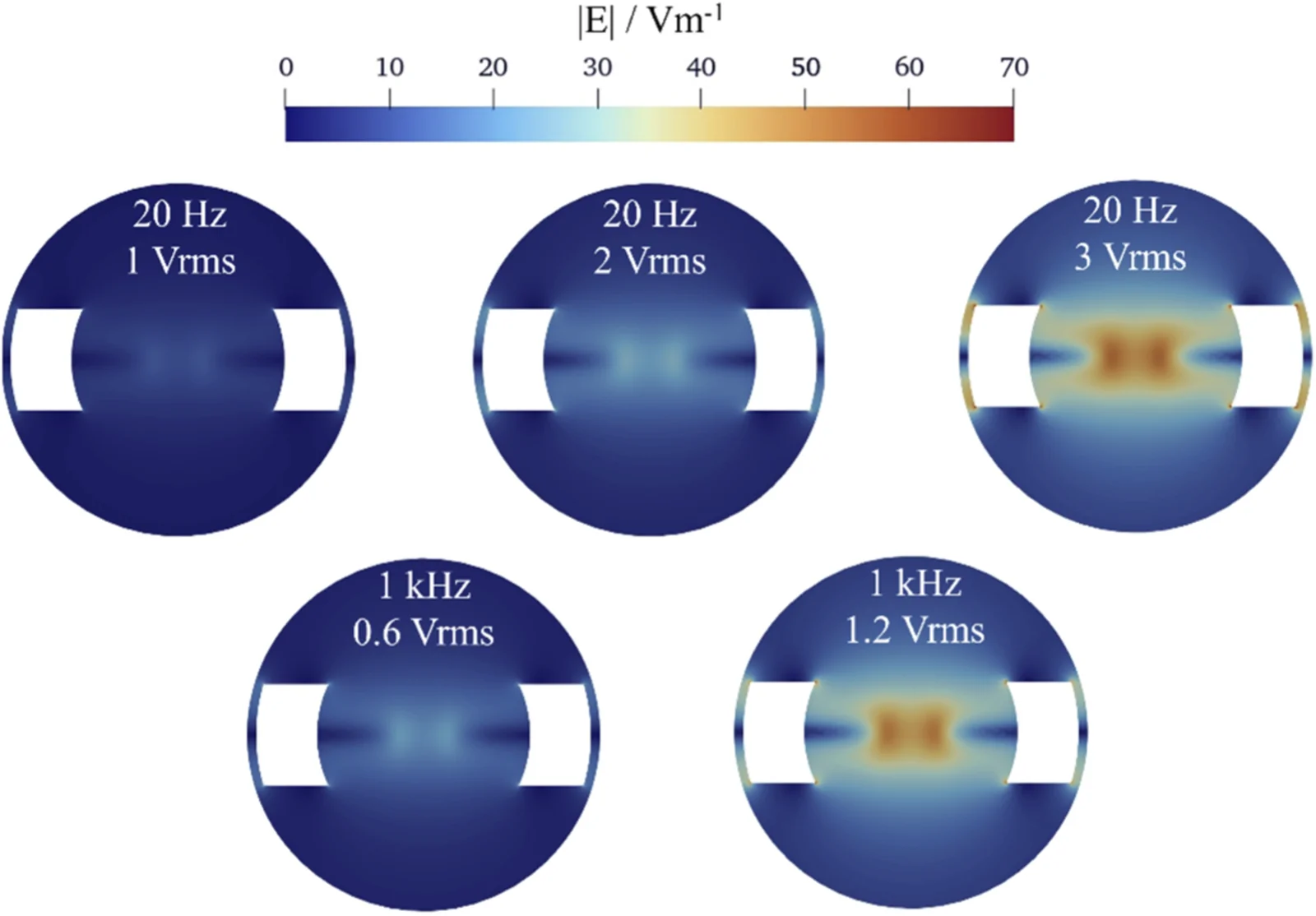
Representative numerically simulated electric field magnitude distributions (Vm^-1^) for hASC stimulation experiments, extracted at a horizontal plane 4 µm above the well bottom (top view). Shown are one representative electrode and time point per applied voltage condition: 20 Hz at 1, 2, and 3 Vrms, and 1 kHz at 0.6 and 1.2 Vrms. Simulations assumed a symmetric impedance distribution and were visualized using ParaView (v6.0.1; Kitware Inc.).

**FIGURE 2:**
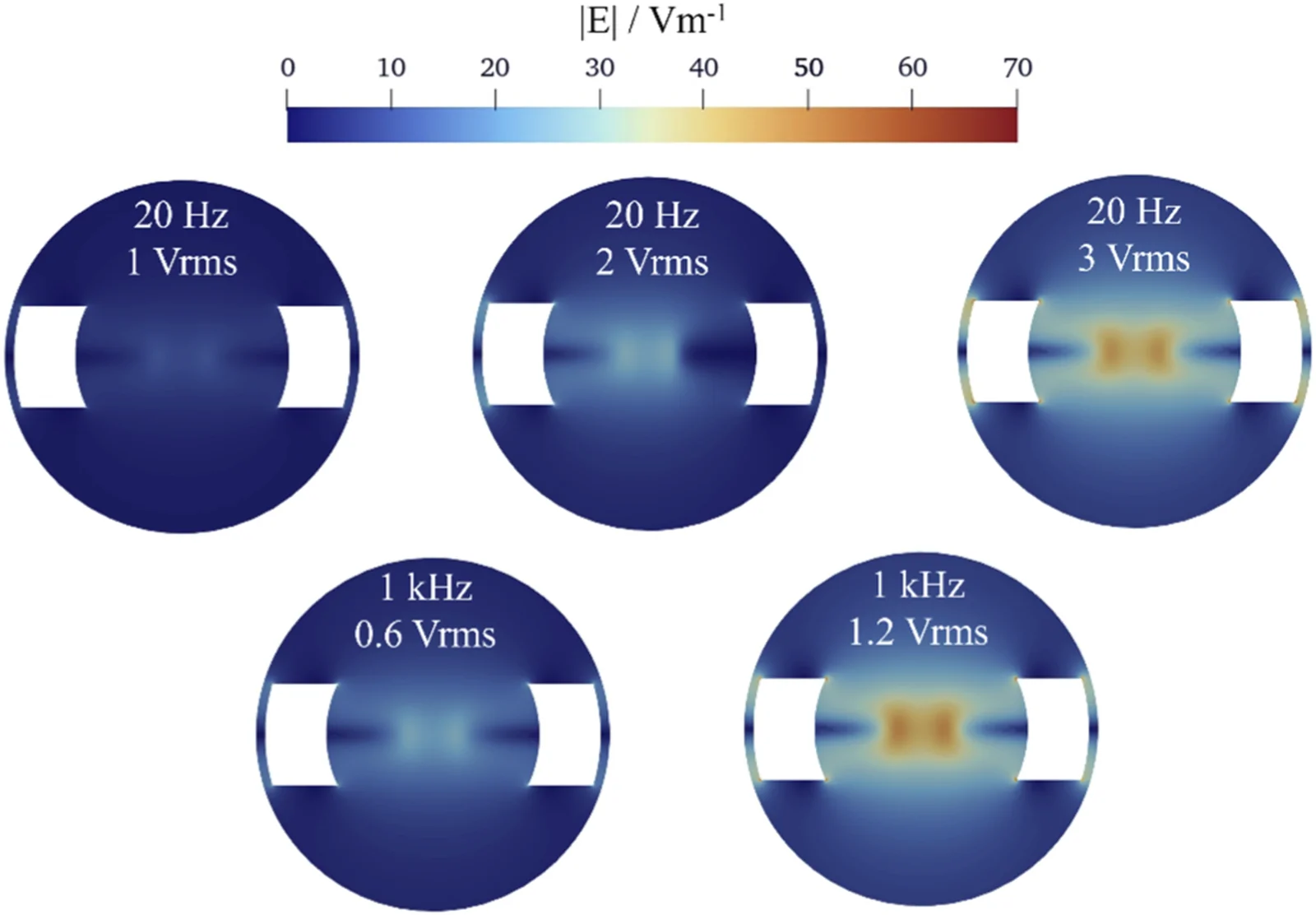
Representative numerically simulated electric field magnitude distributions (Vm^-1^) for hMSC-BM stimulation experiments, extracted at a horizontal plane 4 µm above the well bottom (top view). Shown are one representative electrode and time point per applied voltage condition: 20 Hz at 1, 2, and 3 Vrms, and 1 kHz at 0.6 and 1.2 Vrms. Simulations assumed a symmetric impedance distribution and were visualized using ParaView (v6.0.1; Kitware Inc.).

Representative electric field distributions are shown in Figure 1 for hASC and Figure 2 for hMSC-BM. For each condition, the displayed simulation corresponds to the ES experiment, electrode, and time point whose electric field magnitude at the central evaluation position was closest to the respective temporal mean, thereby providing a representative visualization of the field conditions during the three-day stimulation period.

Representative electric field distributions at 4 µm above the well bottom confirmed the voltage-dependent increase in field intensity observed in the quantitative area analysis. At 20 Hz, 1 Vrms produced predominantly low field magnitudes across the culture surface, whereas 2 Vrms resulted in an expanded central region of moderate field strength. At 3 Vrms, areas exceeding 15 Vm^-1^ became clearly visible between the electrodes, with localized maxima near the electrode boundaries.

A similar spatial pattern was observed at 1 kHz. While 0.6 Vrms was associated mainly with low to moderate field magnitudes, 1.2 Vrms generated an extended region of elevated field strength that visually resembled the distribution observed at 20 Hz and 3 Vrms. In both cell-type-specific simulations, the overall field geometry remained comparable, with symmetric field amplification between the electrodes and localized intensity maxima near the electrode edges.

### 3.2 Effects of alternating electric stimulation on cell viability in stem cells

**FIGURE 3:**
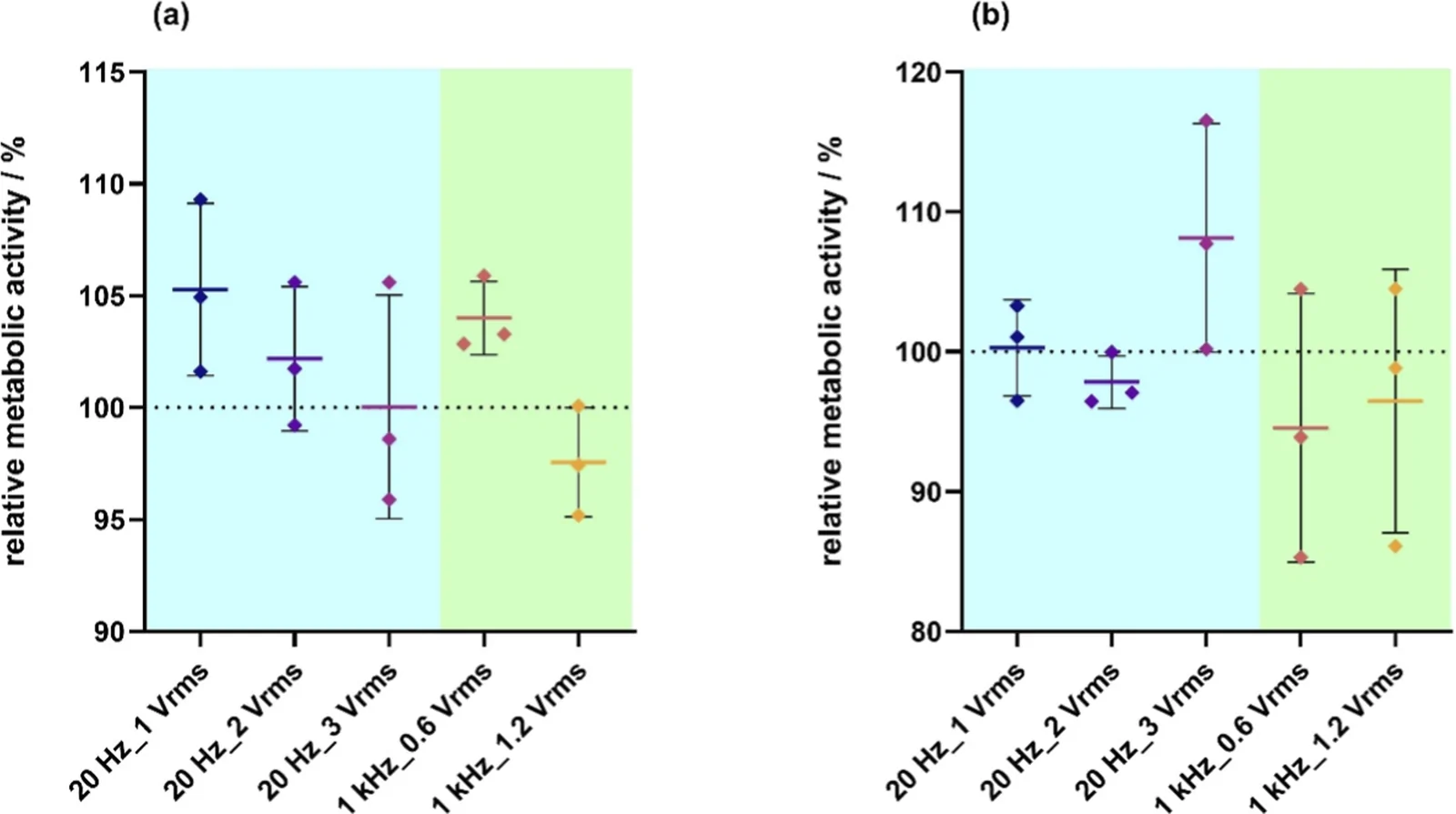
Relative metabolic activity of **(a)** hASC and **(b)** hMSC-BM after 3 days of ES with sinusoidal AC. Cells were stimulated at 20 Hz (1, 2, and 3 Vrms) and 1 kHz (0.6 and 1.2 Vrms). Metabolic activity was normalized to the respective unstimulated control and expressed as percentage. Statistical analysis was performed using one-sample *t*-tests against the normalized reference value of 100%. Non-significant differences are not shown. Data are shown as mean ± SD of technical triplicates (*n* = 3) derived from the same biological cell population.

After three days of ES at 20 Hz (1, 2, and 3 Vrms) and 1 kHz (0.6 and 1.2 Vrms), no statistically significant changes in relative metabolic activity were observed in hASC or hMSC-BM compared with unstimulated controls (Figure 3). Relative metabolic activity ranged from 97.6% to 105.3% in hASC and from 94.6% to 108.1% in hMSC-BM across all stimulation conditions. One-sample *t*-tests against the normalized reference value of 100% revealed no statistically significant deviations for any condition.

Notably, these findings were obtained under spatially inhomogeneous electric field conditions at the cell layer, as demonstrated by the representative numerical field simulations for hASC and hMSC-BM shown in Figures 1 and 2, respectively. Despite heterogeneous local field magnitudes across the culture surface, no detectable reduction in relative metabolic activity was observed. Overall, relative metabolic activity remained stable in both cell types throughout the three-day stimulation period.

### 3.3 Effects of alternating electrical stimulation on early gene expression in stem cells

The effects of ES on early gene expression were analyzed in hASC and hMSC-BM after 3 days of stimulation at 20 Hz (1, 2, and 3 Vrms) and 1 kHz (0.6 and 1.2 Vrms). Gene expression levels were normalized to the reference gene 18S rRNA and calculated relative to the respective unstimulated controls. Importantly, transcriptional responses were evaluated under spatially inhomogeneous electric field conditions at the cell layer, as demonstrated by numerical field simulations in Figures 1 and 2, resulting in heterogeneous local field exposure across the culture surface.

The selected gene panel covered osteogenesis-associated markers (*ALPL*, *COL1A1*, *SPARC*), cellular stress responses and protein folding (*HSPB1*, *HSPA1A*), cell death- and survival-associated mechanisms (*CASP3*, *MLKL*, *GPX4*), as well as cell cycle regulation and proliferation (*CCNB1*, *CDKN1A*, *MKI67*). Results are presented according to these functional gene groups to distinguish early osteogenesis-associated transcriptional changes from stress-, survival-, and proliferation-related responses.

#### 3.3.1 Effects on early osteogenesis-associated gene expression

**FIGURE 4:**
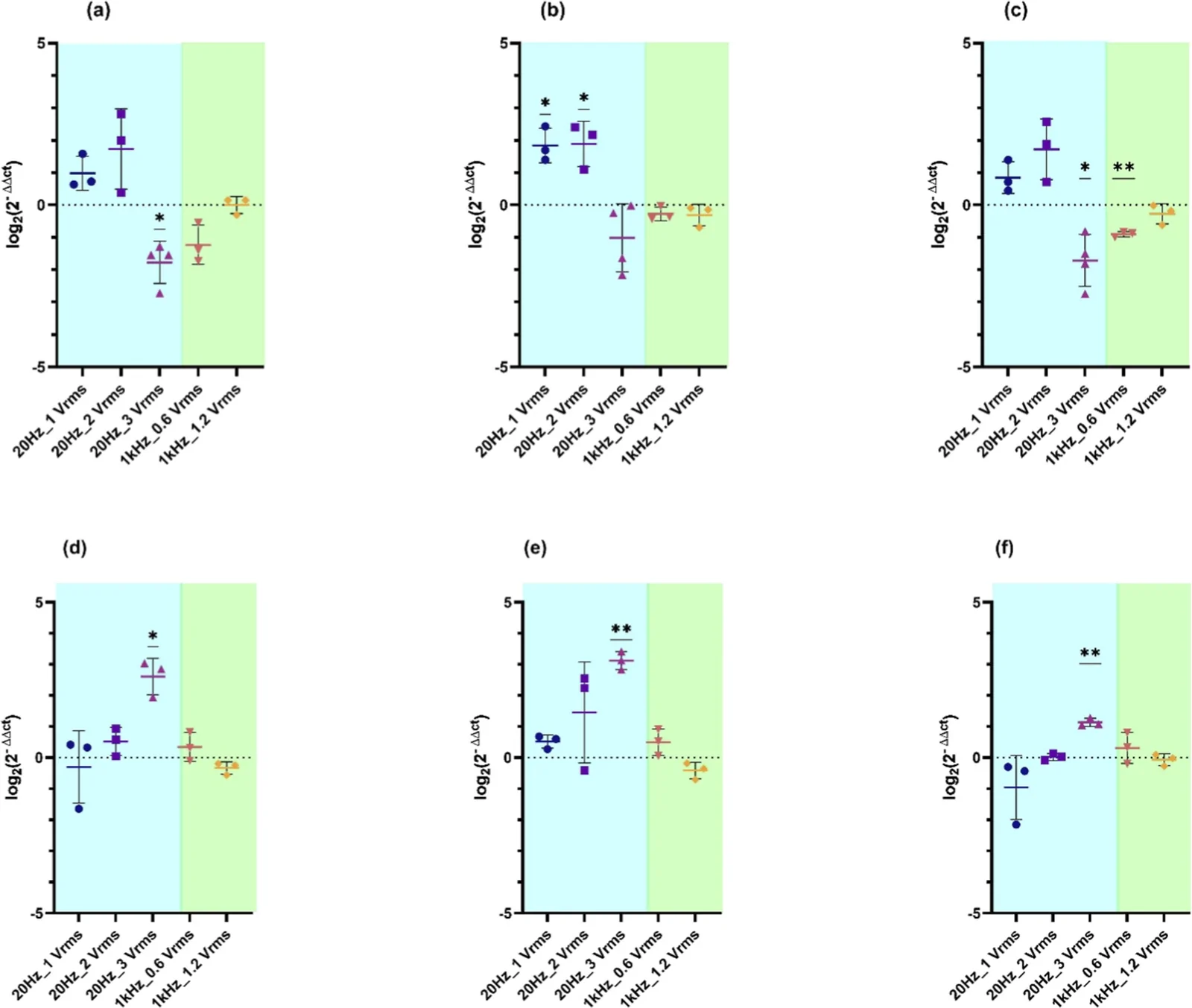
Relative mRNA expression of osteogenesis-associated genes alkaline phosphatase, biomineralization-associated (*ALPL*), collagen type I alpha 1 chain (*COL1A1*), and secreted protein acidic and cysteine-rich (*SPARC*) in hASC **(a–c)** and hMSC-BM **(d–f)** after 3 days of ES. ES was applied as sinusoidal AC at 20 Hz (1, 2, and 3 Vrms) and 1 kHz (0.6 and 1.2 Vrms). Gene expression levels were normalized to the reference gene 18S rRNA, calculated relative to the respective unstimulated control using the 2^−ΔΔCt^ method, and presented as log_2_ (^2−ΔΔCt^). Statistical analysis was performed using one-sample *t*-tests against 0. Non-significant differences are not shown. Individual data points represent technical replicates derived from the same biological cell population; data are shown as mean ± SD (*n* = 3), except for hASC exposed to 3 Vrms at 20 Hz (*n* = 4). *p* < 0.05 (*) and *p* < 0.01 (**).

After 3 days of ES, hASC and hMSC-BM showed cell type-specific, frequency- and voltage-dependent changes in the expression of osteogenesis-associated genes (Figure 4). In hASC, stimulation at 20 Hz induced a mixed transcriptional response. While *COL1A1* was significantly upregulated at 1 Vrms and 2 Vrms, corresponding to 3.6-fold and 3.7-fold increases, respectively, stimulation at 3 Vrms resulted in significant downregulation of *ALPL* and *SPARC*, each by approximately 3.3-fold. In addition, *SPARC* expression was significantly reduced by 1.9-fold under 1 kHz stimulation at 0.6 Vrms.

In hMSC-BM, the most pronounced response was observed at 20 Hz and 3 Vrms. Under this condition, *ALPL*, *COL1A1*, and *SPARC* were significantly upregulated by 6.1-fold, 8.7-fold, and 2.2-fold, respectively. In contrast, no statistically significant changes in osteogenesis-associated gene expression were detected under 1 kHz stimulation. Together, these results indicate that early osteogenesis-associated transcriptional responses to ES were cell type-specific and frequency-dependent, with 20 Hz stimulation inducing detectable changes in both hASC and hMSC-BM. However, the direction and magnitude of these responses differed between cell types and depended on the applied voltage amplitude.

#### 3.3.2 Effects on early expression of genes associated with cellular stress and protein folding

**FIGURE 5:**
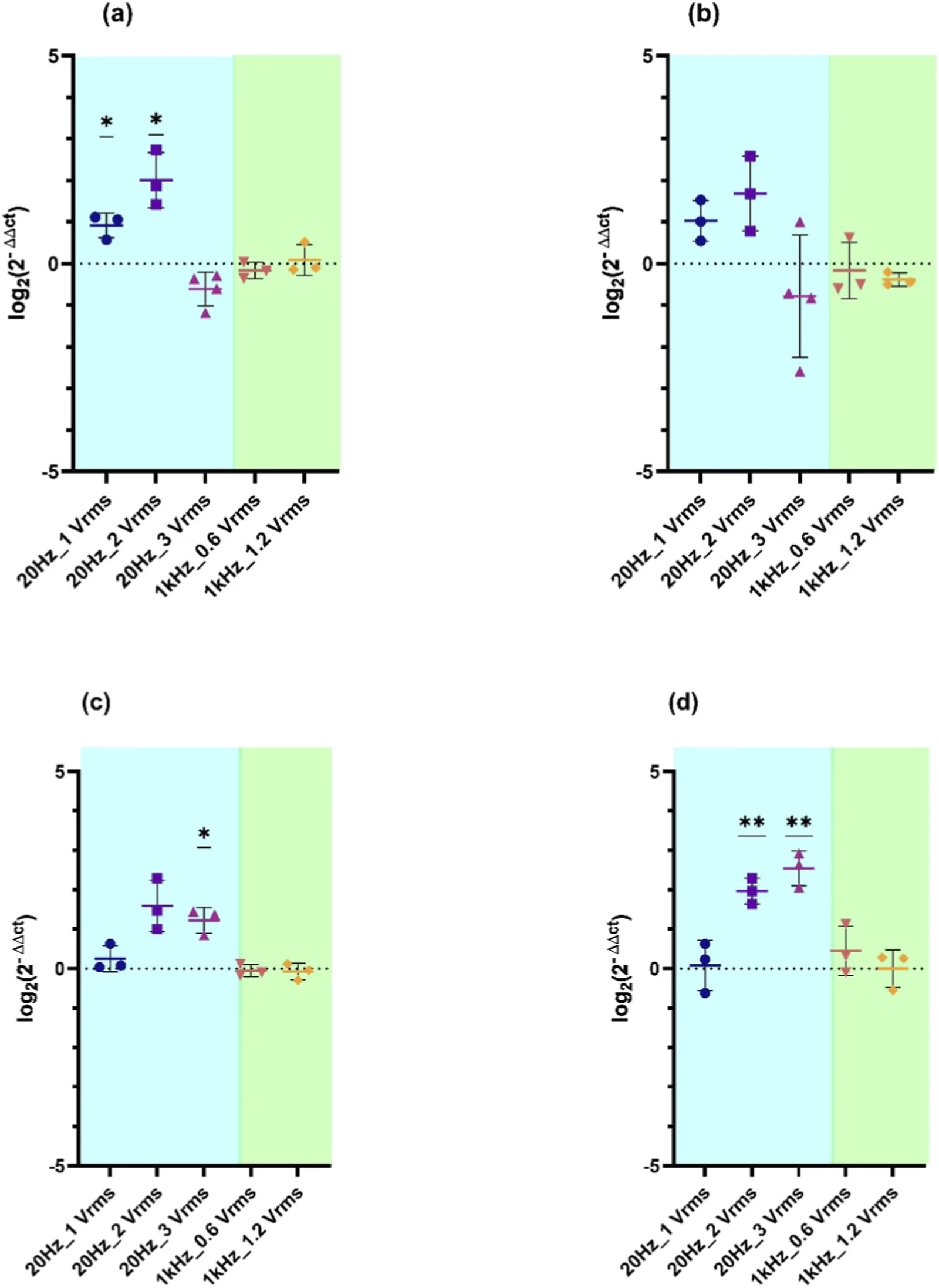
Relative mRNA expression of stress-associated genes involved in cellular stress regulation and protein folding, heat shock protein family B member 1 (*HSPB1*) and heat shock protein family A member 1A (*HSPA1A*), in hASC **(a,b)** and hMSC-BM **(c,d)** after 3 days of ES. ES was applied as sinusoidal AC at 20 Hz (1, 2, and 3 Vrms) and 1 kHz (0.6 and 1.2 Vrms). Gene expression levels were normalized to the reference gene 18S rRNA, calculated relative to the respective unstimulated control using the 2^−ΔΔCt^ method, and presented as log_2_ (2^−ΔΔCt^). Statistical analysis was performed using one-sample *t*-tests against 0. Non-significant differences are not shown. Individual data points represent technical replicates derived from the same biological cell population; data are shown as mean ± SD (*n* = 3), except for hASC exposed to 3 Vrms at 20 Hz (*n* = 4). *p* < 0.05 (*) and *p* < 0.01 (**).

After 3 days of ES, hASC and hMSC-BM showed cell type-specific, frequency- and voltage-dependent changes in the expression of stress-associated genes (Figure 5). In hASC, stimulation at 20 Hz induced significant upregulation of *HSPB1* at 1 Vrms and 2 Vrms, corresponding to 1.9-fold and 4.0-fold increases, respectively. In contrast, no statistically significant changes in *HSPA1A* expression were detected in hASC under any stimulation condition.

In hMSC-BM, significant upregulation of stress-associated genes was mainly observed under 20 Hz stimulation. *HSPB1* expression was significantly increased by 2.3-fold at 3 Vrms. *HSPA1A* expression was significantly upregulated at 2 Vrms and 3 Vrms, corresponding to 3.9-fold and 5.9-fold increases, respectively. No statistically significant changes in *HSPB1* or *HSPA1A* expression were observed under 1 kHz stimulation. Together, these findings indicate that early stress-associated transcriptional responses were primarily induced by 20 Hz stimulation.

#### 3.3.3 Effects on early expression of genes involved in cell death and survival mechanisms

**FIGURE 6:**
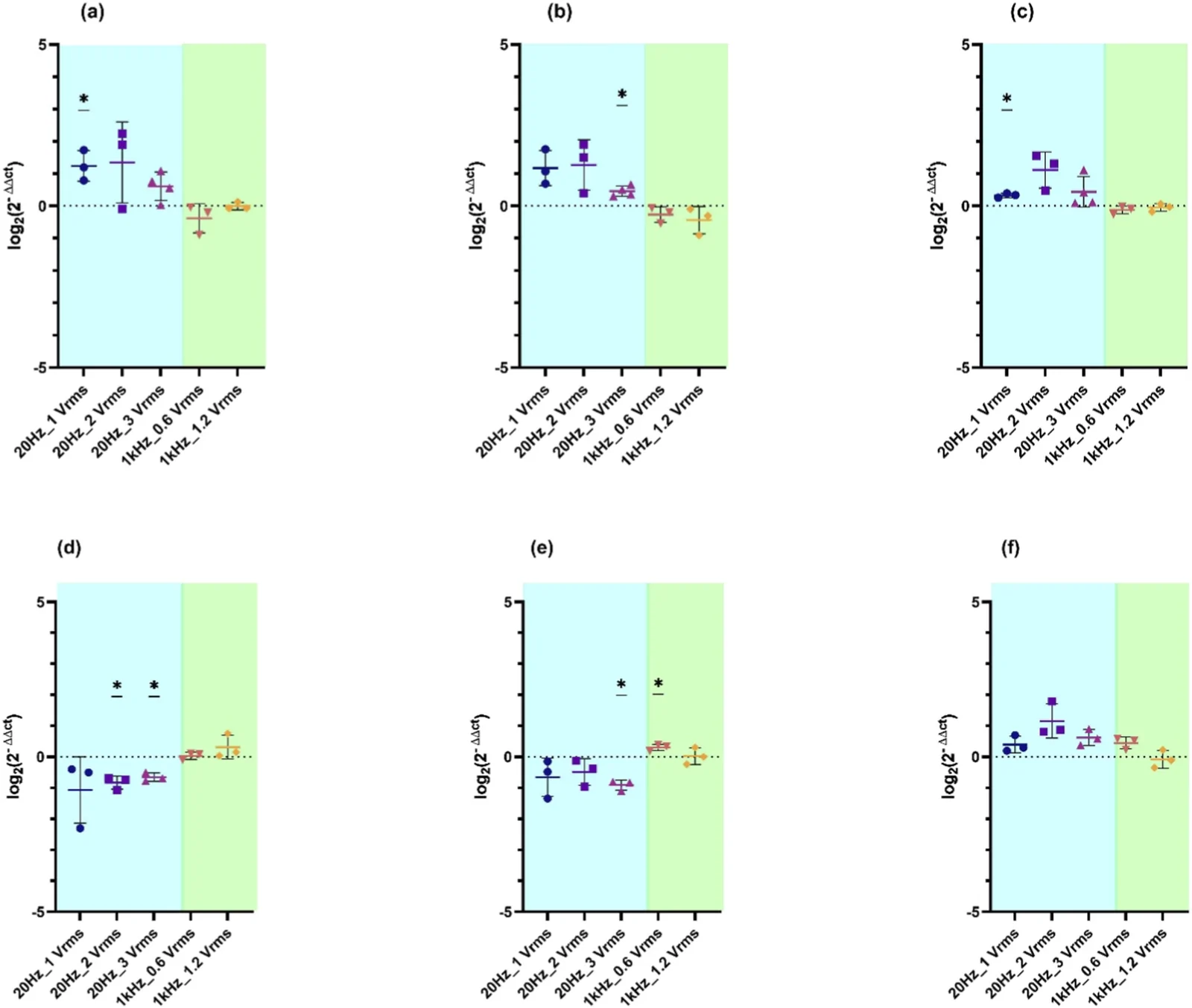
Relative mRNA expression of cell death- and survival-associated genes caspase 3 (*CASP3*), mixed lineage kinase domain-like pseudokinase (*MLKL*), and glutathione peroxidase 4 (*GPX4*) in hASC **(a–c)** and hMSC-BM **(d–f)** after 3 days of ES. ES was applied as sinusoidal AC at 20 Hz (1, 2, and 3 Vrms) and 1 kHz (0.6 and 1.2 Vrms). Gene expression levels were normalized to the reference gene 18S rRNA, calculated relative to the respective unstimulated control using the 2^−ΔΔCt^ method, and presented as log_2_ (2^−ΔΔCt^). Statistical analysis was performed using one-sample *t*-tests against 0. Non-significant differences are not shown. Individual data points represent technical replicates derived from the same biological cell population; data are shown as mean ± SD (*n* = 3), except for hASC exposed to 3 Vrms at 20 Hz (*n* = 4). *p* < 0.05 (*).

After 3 days of ES, hASC and hMSC-BM showed cell type-specific, frequency- and voltage-dependent changes in the expression of genes associated with regulated cell death and survival pathways (Figure 6). In hASC, *CASP3* expression was significantly upregulated by 2.3-fold at 20 Hz and 1 Vrms. *MLKL* expression was significantly increased by 1.4-fold at 20 Hz and 3 Vrms, while *GPX4* expression was significantly upregulated at 20 Hz and 1 Vrms. No statistically significant changes in these genes were detected under 1 kHz stimulation in hASC.

In hMSC-BM, *CASP3* expression was significantly reduced under 20 Hz stimulation at 2 Vrms and 3 Vrms. *MLKL* expression was significantly reduced at 20 Hz and 3 Vrms and additionally at 1 kHz and 0.6 Vrms. In contrast, *GPX4* expression remained unchanged under all stimulation conditions.

#### 3.3.4 Effects on early cell cycle- and proliferation-associated gene expression

**FIGURE 7:**
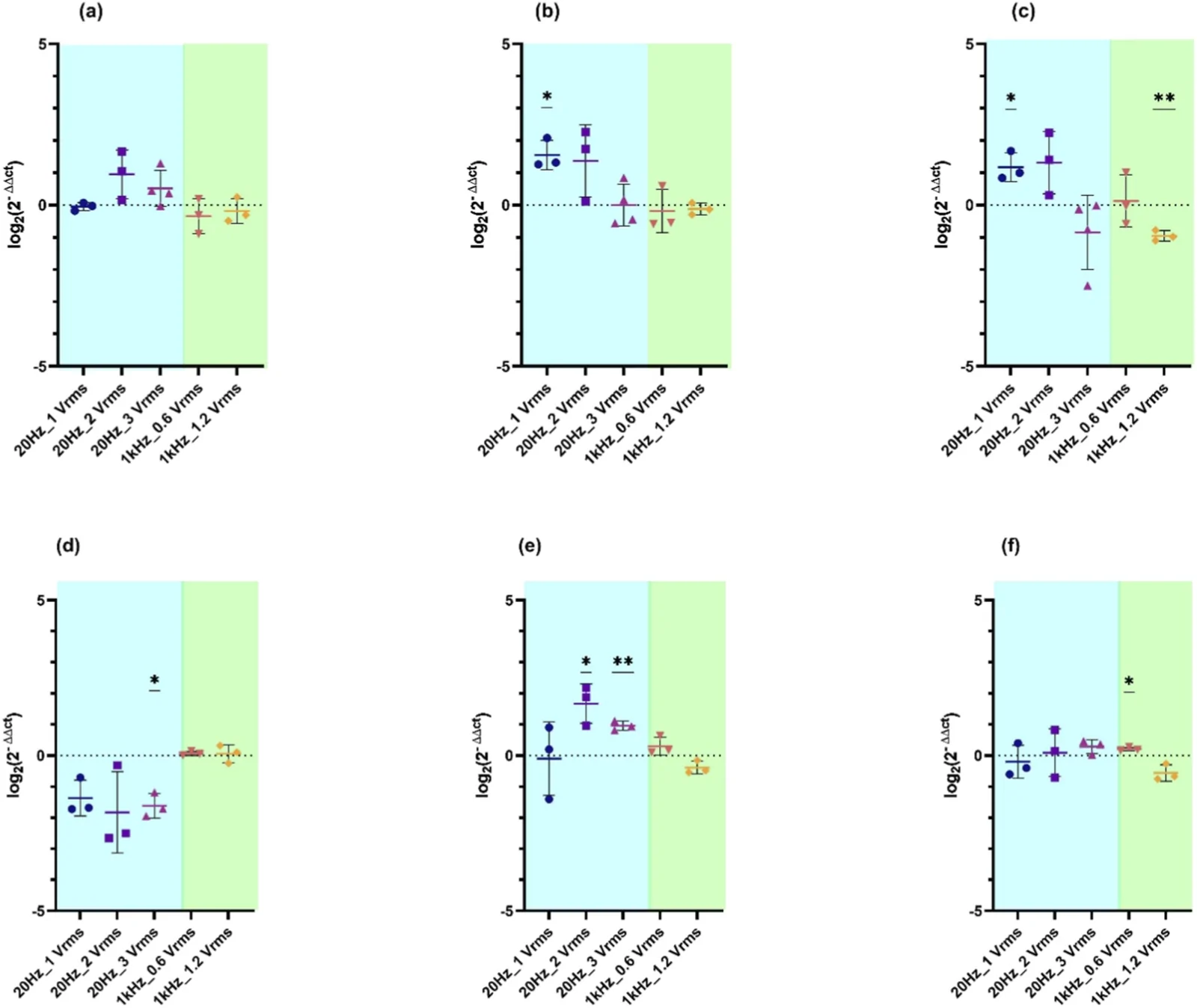
Relative mRNA expression of cell cycle- and proliferation-associated genes cyclin B1 (*CCNB1*), cyclin-dependent kinase inhibitor 1A (*CDKN1A*), and marker of proliferation Ki-67 (*MKI67*) in hASC **(a–c)** and hMSC-BM **(d–f)** after 3 days of ES. ES was applied as sinusoidal AC at 20 Hz (1, 2, and 3 Vrms) and 1 kHz (0.6 and 1.2 Vrms). Gene expression levels were normalized to the reference gene 18S rRNA, calculated relative to the respective unstimulated control using the 2^−ΔΔCt^ method, and presented as log_2_ (^2−ΔΔCt^). Statistical analysis was performed using one-sample *t*-tests against 0. Non-significant differences are not shown. Individual data points represent technical replicates derived from the same biological cell population; data are shown as mean ± SD (*n* = 3), except for hASC exposed to 3 Vrms at 20 Hz (*n* = 4). *p* < 0.05 (*) and *p* < 0.01 (**).

After 3 days of ES, hASC and hMSC-BM showed cell type-specific, frequency- and voltage-dependent changes in the expression of genes associated with cell cycle regulation and proliferation (Figure 7). In hASC, *CCNB1* expression remained unchanged under all stimulation conditions. *CDKN1A* expression was significantly upregulated by 2.9-fold at 20 Hz and 1 Vrms. *MKI67* expression was significantly increased by 2.3-fold at 20 Hz and 1 Vrms, whereas it was significantly reduced by 1.9-fold at 1 kHz and 1.2 Vrms.

In hMSC-BM, *CCNB1* expression was significantly reduced by approximately 3-fold at 20 Hz and 3 Vrms. *CDKN1A* expression was significantly increased at 20 Hz, corresponding to 3.3-fold and 1.9-fold increases at 2 Vrms and 3 Vrms, respectively. *MKI67* expression was significantly upregulated at 1 kHz and 0.6 Vrms, while no statistically significant changes were detected under 20 Hz stimulation.

## 4 Discussion

The electrical characterization of the stimulation system provides an essential basis for interpreting the observed biological responses, because nominal voltage alone does not reflect the effective electrical conditions experienced by adherent cells. In the present study, measurements performed during 3 days of sinusoidal AC-ES revealed a pronounced voltage-dependent decrease in impedance at 20 Hz, whereas impedance at 1 kHz remained largely stable across the applied voltage range. This frequency-dependent behavior is consistent with enhanced electrode polarization and increased electrochemical contributions at lower frequencies, as described previously (Franks et al., 2005; McAdams and Jossinet, 1994). The central objective of the present study was therefore not to demonstrate terminal osteogenic differentiation, but to relate measured electrical system characteristics and cell-layer-resolved electric-field exposure to early biological response patterns. This provides a methodological bridge between nominal stimulation settings and biologically interpretable exposure conditions, which is required before selected conditions can be examined in longer-term and functional validation experiments.

Both culture media employed in this study were selected to support optimal growth and viability of the respective stem-cell populations. Importantly, the ES parameters applied in hASC- and hMSC-BM-specific experiments were closely comparable with respect to current amplitudes, impedance behavior, and resulting electric-field strengths, thereby supporting a comparative interpretation of frequency-dependent transcriptional responses between both cell populations. Nevertheless, differences in the biochemical composition of the culture media may have contributed to cell-population-associated or condition-dependent responses under ES. Medium-mediated electrochemical effects have been reported for current-controlled 20 Hz AC-ES systems, particularly under conditions of increased electrode polarization and elevated interfacial driving voltages (Bielfeldt et al., 2023). Such effects should therefore be considered when interpreting frequency- and amplitude-dependent cellular responses, especially at low stimulation frequencies.

In the present work, continuous sinusoidal AC-ES was primarily performed within parameter regimes previously shown to remain stable and reproducible at 20 Hz, namely 1 and 2 Vrms. In a prior study, stimulation at 20 Hz and 3 Vrms was associated with progressive changes at the EEI during prolonged exposure, including indications of salt crystallization, whereas 1 and 2 Vrms remained stable over a three-day stimulation period (Lembcke et al., 2026). Based on these findings, 1 and 2 Vrms at 20 Hz were considered comparatively stable stimulation conditions within this system. Stimulation at 20 Hz and 3 Vrms was nevertheless intentionally included as the highest tested continuous amplitude to assess cellular responses under increased electrical and interfacial loading. It should therefore be regarded as an exploratory condition within the present system rather than as a generally transferable stimulation limit.

In contrast to *in vivo* electrostimulation concepts, where empirically derived tissue-level electrical gradients have been discussed in the context of bone regeneration (Kraus, 1984) and applied in implant-based approaches (Kämmerer et al., 2025; Raben et al., 2019), no standardized electric-field range exists for *in vitro* stem-cell stimulation (Meng et al., 2022; Ryan et al., 2021). Consequently, stimulation intensity is often defined by nominal voltage rather than by quantified cell-level electric-field exposure. This is particularly limiting in direct electrode-based systems, where EEI effects, impedance behavior, and medium conductivity can substantially influence the effective field distribution experienced by adherent cells (Zimmermann et al., 2023). Moreover, implant-related numerical models have shown that electrode–tissue interface parameters and tissue dielectric properties can substantially influence simulated field distributions (Raben et al., 2024). Accordingly, parameter ranges discussed for implant-associated or tissue-level applications cannot be transferred directly to the present cell-culture system.

Building on the experimentally validated framework described by Lembcke et al. (2026), electric-field distributions in the present study were calculated using FE simulations parameterized with experimentally determined electrical data, including effective voltage, impedance magnitude, and phase behavior (Lembcke et al., 2026). By incorporating EEI-related boundary conditions, the simulations provided a cell-layer-resolved estimate of electric-field exposure at 4 µm above the culture surface. Rather than relying on a single averaged field value, field magnitudes were stratified into defined exposure ranges, allowing biological responses to be interpreted in relation to spatially resolved and experimentally grounded stimulation conditions.

The simulations confirmed that increasing stimulation amplitude redistributed the exposed culture area across defined electric-field ranges, with higher amplitudes increasing the proportion of the cell layer exposed to moderate and higher field magnitudes. This redistribution was observed at both 20 Hz and 1 kHz, although the underlying electrical behavior differed between frequencies. Thus, the effective stimulation condition cannot be adequately described by applied voltage alone, but depends on the combined influence of frequency, impedance, electrode–medium coupling, and local field distribution. By providing EEI-aware electric-field quantification based on measured electrical parameters, the present approach addresses a key limitation in *vitro* ES studies, namely, insufficient transparency of cell-level field characterization (Budde et al., 2019; Zimmermann et al., 2021; Zimmermann et al., 2023).

This system-specific approach is consistent with broader concepts in regenerative medicine, in which biophysical cues are considered alongside cells, biomaterials, and biochemical factors. Hao et al. (2021) described biophysical stimuli as an additional component of bone tissue engineering and emphasized that osteoinductive effects depend on the respective physical stimulus and its application conditions (Hao et al., 2021). Similarly, approaches using electroactive scaffolds, piezoelectric materials, or self-powered systems aim to reproduce aspects of bioelectric microenvironments in regenerative settings (Hao et al., 2025). Although these approaches use different modes of electrical coupling and material interfaces, they share the aim of relating defined biophysical cues to cellular responses within a controlled regenerative microenvironment. They therefore provide a complementary perspective for the present work and underscore that the biological interpretation of ES benefits from physical characterization within the respective experimental system.

As metabolic activity was assessed using a bulk XTT readout normalized to the respective unstimulated control, this endpoint provides an overall estimate of relative metabolic activity after the three-day stimulation period. Under the applied stimulation conditions, relative metabolic activity remained close to control levels, indicating no detectable impairment of the bulk metabolic readout, although localized cellular effects within the spatially heterogeneous electric field cannot be excluded. The absence of a marked metabolic decline is consistent with previous *in vitro* studies showing that moderate AC-ES can induce regulatory or differentiation-associated gene-expression changes in human stem and bone-related cells without major effects on proliferation or metabolic activity (Hlavac et al., 2021; Sahm et al., 2020). In contrast, reduced viability has been reported under substantially higher stimulation intensities using low-frequency pulsed protocols with nominal field strengths of up to approximately 800 Vm^-1^ (Beugels et al., 2019). Although these stimulation paradigms are not directly comparable with respect to waveform, coupling mode, exposure duration, and effective field estimation, they support the general concept that excessive electrical amplitudes may compromise cellular metabolic stability. The XTT assay was included as a short-term compatibility screen to support the interpretation of subsequent molecular readouts under conditions without an apparent reduction in relative bulk metabolic activity. However, localized, transient, or non-metabolic cellular effects cannot be excluded.

The effects of ES on early transcriptional responses were evaluated in hASC and hMSC-BM after 3 days of stimulation across defined voltage and frequency regimes. By interrogating a targeted gene panel covering osteogenesis-associated markers, cellular stress regulation, cell death- and survival-associated pathways, and cell-cycle control in two human stem-cell populations of different tissue origin, the present work provides a comparative framework for interpreting parameter-dependent transcriptional responses. However, as the analyses were performed using technical replicates from the same biological cell population, these findings should be considered exploratory and require validation in future studies using independent biological replicates.

Gene expression was normalized to 18S rRNA, which has been shown to exhibit stable expression under comparable ES conditions and stimulation durations, particularly at stimulation amplitudes exceeding 2 Vrms (Lembcke et al., 2026). Future studies may further improve normalization robustness by implementing multi-reference strategies incorporating additional housekeeping genes, such as *YWHAZ* and *PPIA*.

The osteogenesis-associated gene-expression patterns observed in the present study reflect early, parameter-dependent transcriptional responses to AC-ES and are consistent with previous *in vitro* reports describing defined stimulatory windows for electrical modulation. In hASC, moderate stimulation at 20 Hz between one and 2 Vrms was associated with increased *COL1A1* expression, whereas 3 Vrms at 20 Hz was associated with reduced *ALPL* and *SPARC* transcript levels. In hMSC-BM, the most pronounced transcriptional response was observed at 20 Hz and 3 Vrms, with increased expression of *ALPL*, *COL1A1*, and *SPARC*. These findings indicate differences between the investigated cell populations under the applied conditions, but they do not establish general cell-type-specific response patterns because only one biological cell population per stem-cell type was analyzed.

The transition from increased to reduced expression of selected osteogenesis-associated markers at higher amplitudes is consistent with observations in osteoblast-like cells, where 20 Hz AC stimulation at moderate voltages enhanced osteogenic marker expression, whereas higher amplitudes induced a time-dependent downregulation of *COL1A1*, *ALP*, and *BGLAP*, particularly in cells cultured directly on electrodes (Sahm et al., 2020). Early structural evidence for such a shift was provided by Wiesmann et al. (2000), who demonstrated that capacitively coupled ES of osteoblast-like cells enhanced physiological biomineralization, whereas higher effective voltage drops under semi-capacitive coupling resulted in aberrant, non-physiological mineral precipitation (Wiesmann et al., 2000). In line with this concept, Creecy et al. (2013) showed that AC stimulation at 20 Hz promoted osteogenic differentiation of human MSCs in a time- and amplitude-dependent manner, while adipogenic and chondrogenic markers remained absent (Creecy et al., 2013). Comparable principles have also been reported for direct-current-based stimulation paradigms, where moderate ES enhanced osteogenic differentiation and bone formation *in vitro* and *in vivo* (Leppik et al., 2018; Mobini et al., 2016).

Together, these findings and the present data suggest that, within this stimulation setup and for the investigated cell populations, early osteogenesis-associated transcriptional responses are modulated by both amplitude and frequency. Under the applied conditions, hASC showed reduced expression of selected osteogenesis-associated markers at higher AC amplitudes, whereas hMSC-BM showed increased expression of these markers under comparable stimulation conditions. Importantly, these results do not demonstrate ES-induced terminal osteogenic differentiation. They instead identify response patterns that may guide the selection of conditions for subsequent analyses of alkaline phosphatase activity, protein expression, extracellular matrix deposition, and mineralization.

Beyond osteogenesis-associated gene expression, the present data indicate that AC-ES may modulate stress-associated transcriptional responses in a frequency- and voltage-dependent manner. Changes in *HSPB1* and *HSPA1A* expression were observed primarily under 20 Hz stimulation and were not detected at 1 kHz, suggesting that low-frequency stimulation may induce selective stress-associated gene-expression patterns rather than a generalized impairment of cellular metabolic activity. Heat-shock proteins are central mediators of cellular stress adaptation and have been implicated in maintaining stem-cell functionality under challenging environmental conditions (Shende et al., 2019). The selection of *HSPB1* and *HSPA1A* was further guided by Hronik-Tupaj et al. (2011), who investigated osteogenic differentiation and stress-associated responses in hMSC-BM exposed to AC electric fields (Hronik-Tupaj et al., 2011). In their long-term model, cells were cultured under osteogenic conditions and exposed to a 60 kHz electric field of 2 Vm^-1^ for 40 min daily over 28 days. In addition to *ALP* and *COL1*, the authors assessed HSP27 and HSP70 expression together with changes in cell morphology, metabolic and redox-associated parameters, collagen deposition, and calcium staining. HSP27 expression increased from day 10 onwards and preceded the later increase in *ALP* and *COL1* expression, whereas HSP70 showed a less consistent temporal pattern and increased only at the later stage of the experiment. The authors therefore proposed a potential relationship between ES, stress-associated regulation, and osteogenic differentiation, while emphasizing that the underlying mechanisms remained unresolved (Hronik-Tupaj et al., 2011).

In a broader context, physical stimuli such as mechanical loading can activate stress-responsive pathways that contribute to osteogenic adaptation (Stewart et al., 2020), and stress-associated mediators, including osteopontin, participate in mechanically induced bone formation (Morinobu et al., 2003). Recent mechanically active culture platforms similarly demonstrate that cellular responses may differ substantially between low and high physical loading conditions (Im et al., 2023; 2025). These systems are not directly comparable with the present electrical setup, but they reinforce the general principle that the biological consequences of a physical stimulus depend on its magnitude, exposure regimen, and the physical properties of the experimental platform. ES has likewise been reported to induce controlled oxidative stress responses under defined conditions (Bielfeldt et al., 2022; 2023). Within this framework, the voltage-dependent changes in *HSPB1* and *HSPA1A* observed here suggest that AC-ES may influence stress-associated transcriptional regulation. However, their functional relevance for osteogenesis-associated responses remains unclear and cannot be conclusively determined within the scope of the present work.

Genes associated with cell death, survival, and cell-cycle regulation were included to capture early shifts in cellular homeostasis beyond lineage-associated responses. Such pathways in mesenchymal stem cells are known to respond dynamically to moderate stress without necessarily inducing cell death (Akanyibah et al., 2025). In particular, alterations in *CCNB1*, *CDKN1A*, and *MKI67* have been linked to adaptive survival–proliferation balance rather than irreversible growth arrest, as demonstrated in hMSC-BM cells under hypoxic stress (Wang et al., 2014). In the present study, AC-ES induced only moderate and marker-specific transcriptional modulation, predominantly at 20 Hz, without consistent activation of apoptotic or proliferative programs. While ES has been reported to affect apoptosis and proliferation mainly in direct-current-based systems (Leppik et al., 2020; Love et al., 2017), comparable data for AC-ES remain limited. The observed changes should therefore be interpreted with caution and not equated with functional activation of the respective pathways.

The three-day continuous stimulation period served as a short-term assessment window for comparing early molecular responses across the investigated parameter space. It was not intended to establish an optimal duration for later differentiation outcomes. Time is a further component of the electrical exposure regimen, alongside amplitude, frequency, waveform, field distribution, and stimulation schedule. Previous studies using continuous 20 Hz AC stimulation of hASC across different exposure periods further indicate that cellular outcomes can change over time (Kämmerer et al., 2020). Thus, the present findings can inform subsequent time-course experiments, including longer-term or intermittent stimulation protocols, but these regimens require independent validation.

The stimulation parameters were selected to cover a broad range of electrical exposure, including 20 Hz at 3 Vrms as the highest tested condition in the present system (Lembcke et al., 2026). At 20 Hz, increasing voltage shifted cell-layer exposure from predominantly below 5 Vm^-1^ at 1 Vrms towards higher field ranges at 2 and 3 Vrms. This shift was accompanied by more pronounced transcriptional changes, particularly in hMSC-BM. However, at 3 Vrms, hASC showed reduced expression of selected osteogenesis-associated markers, suggesting that increased electrical and interfacial loading may be perceived differently by the cell populations investigated.

Overall, 20 Hz induced more pronounced transcriptional effects than 1 kHz within the tested parameter space. The 20 Hz, 2 Vrms condition, characterized by increased exposure within the 5–15 Vm^-1^ range, may therefore represent a relevant candidate condition for further investigation, particularly in hMSC-BM. This does not imply that the range is optimal or universally applicable, but that it provides a physically and biologically defined starting point for subsequent validation studies.

Several limitations should be considered. Although two human stem-cell populations from different tissue sources were investigated, each cell type was represented by a single biological cell population. Donor-dependent variability, which can affect MSC expansion, metabolism, morphology, and functional properties, was therefore not addressed and should be included in future studies using independent biological replicates (Heathman et al., 2016; Hwang et al., 2009). In addition, the analyses were restricted to mRNA-level readouts after 3 days of stimulation. The observed patterns therefore represent early transcriptional responses and require confirmation by protein-level analyses and functional assays. Finally, temperature and pH were not continuously monitored during ES. Because electrode-based stimulation can be influenced by electrochemical interface processes and medium-dependent effects, future studies should include real-time or endpoint assessment of temperature and pH to further exclude medium-mediated or electrochemical side effects during stimulation (Bielfeldt et al., 2022; 2023; Franks et al., 2005; Kulkarni et al., 2026).

**FIGURE 8:**
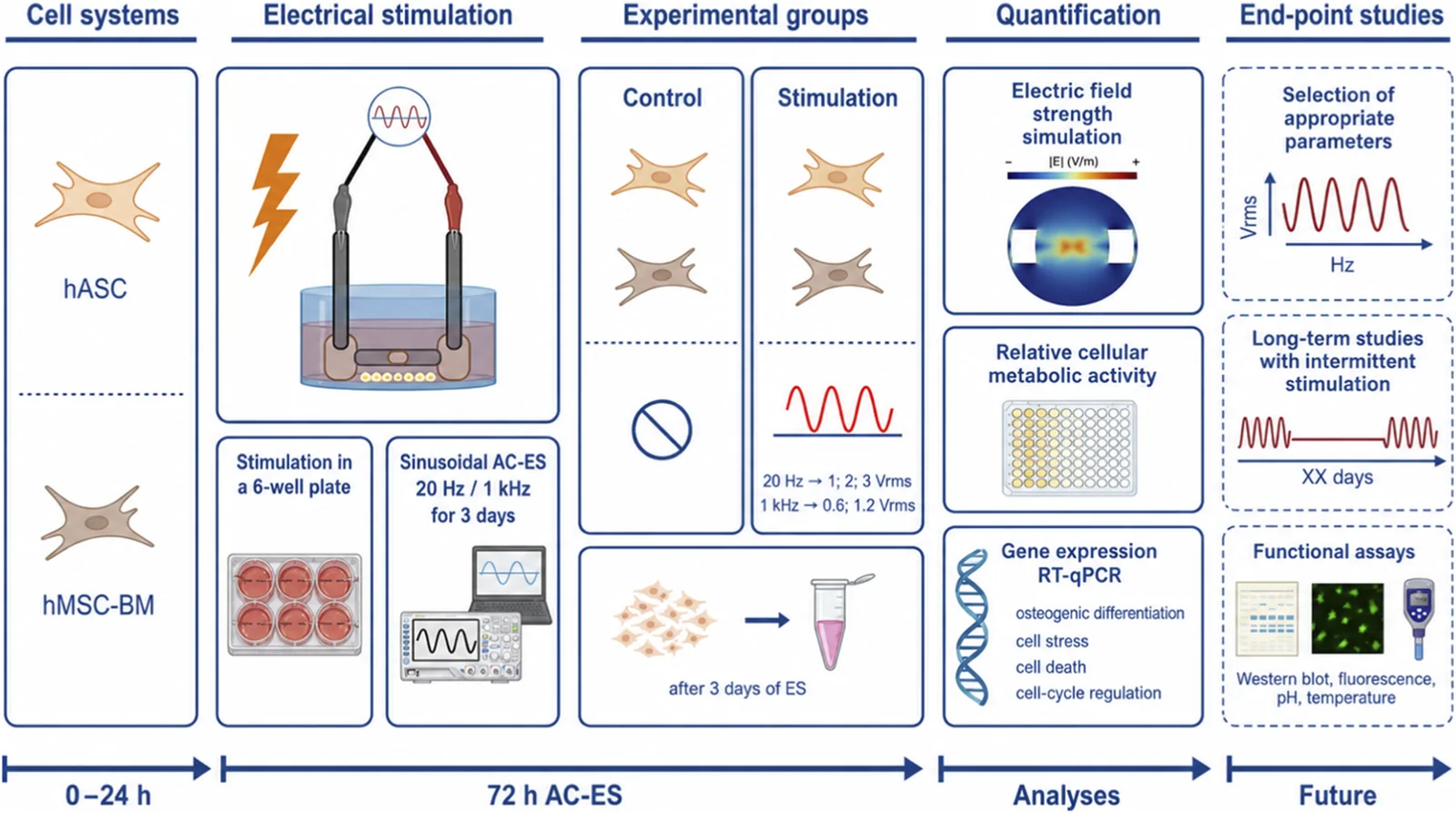
Parameter-resolved AC-ES workflow linking electrical system characteristics to early transcriptional responses in human stem cells. Human adipose-derived stem cells (hASC) and human bone marrow-derived mesenchymal stem cells (hMSC-BM) were exposed to continuous sinusoidal alternating-current electrical stimulation (AC-ES) for 3 days and compared with unstimulated controls. Stimulation was applied at 20 Hz with amplitudes of 1, 2, and 3 Vrms and at 1 kHz with amplitudes of 0.6 and 1.2 Vrms. The stimulation conditions were physically characterized by electrical measurements and numerical electric-field simulations and linked to biological readouts, including relative cellular metabolic activity and RT-qPCR-based assessment of osteogenesis-associated markers, cellular stress responses, regulated cell death and survival-associated pathways, and cell-cycle control and proliferation. The resulting parameter-resolved framework supports the rational selection of stimulation conditions and provides a basis for subsequent studies using independent biological replicates, extended or intermittent stimulation protocols, protein-level analyses, functional assays, and more complex culture models. Created in https://BioRender.com.

Taken together, the present study provides a quantitatively characterized framework for relating defined AC-ES parameters to early transcriptional responses in human stem cells. By systematically varying voltage and frequency and combining EEI-aware, experimentally parameterized electric-field simulations with cell-layer-resolved field stratification, the data support a more transparent interpretation of parameter-dependent responses under defined *in vitro* stimulation conditions (Budde et al., 2019; Lembcke et al., 2026; Zimmermann et al., 2021; 2023). Rather than defining universal stimulation thresholds or demonstrating terminal osteogenic differentiation, the present workflow enables early gene-expression patterns in hASC and hMSC-BM to be interpreted in relation to measured electrical parameters and spatially resolved electric-field exposure. This provides a rational basis for the next experimental stage: validation in independent biological replicates, extended protein- and function-based analyses, time-resolved studies, and ultimately more complex implant-associated models. The parameter-resolved experimental workflow and the resulting directions for subsequent validation studies are summarized in Figure 8.

## 5 Conclusion

This study establishes a parameter-resolved workflow linking measured electrical system characteristics and cell-layer electric-field exposure to early transcriptional responses during sinusoidal AC-ES. Within the investigated system, 20 Hz induced more pronounced osteogenesis- and stress-associated gene-expression changes than 1 kHz, while the 2 cell populations showed different response patterns at higher amplitudes. By integrating EEI-aware electrical characterization with finite-element simulations, the approach provides a reproducible basis for selecting and comparing AC-ES conditions in future studies.

## Data Availability

The original contributions presented in the study are included in the article/Supplementary Material and are additionally available via Zenodo at https://doi.org/10.5281/zenodo.18466464. Further inquiries can be directed to the corresponding author.

## Supplementary material

SUPPLEMENTARY FIGURE 1 Osteogenic differentiation capacity of hASC and hMSC-BM.

SUPPLEMENTARY MATERIAL 1 Summary of electrical stimulation measurements and numerical electric field simulations.

SUPPLEMENTARY MATERIAL 2 Statistical analyses of gene expression data, including normality testing, one-sample *t-*tests, and one-way ANOVA with Tukey’s multiple comparisons test.
