# Supplementary material for: Parameter-resolved AC electrical stimulation links electrical system characteristics to early transcriptional responses in human stem cells
Source: Front Bioeng Biotechnol. 2026 Aug 25;14:1897534. doi: 10.3389/fbioe.2026.1897534 (PMC13550007; doi:10.3389/fbioe.2026.1897534)
Supplement: Supplementary file 1 [file DataSheet1.pdf]

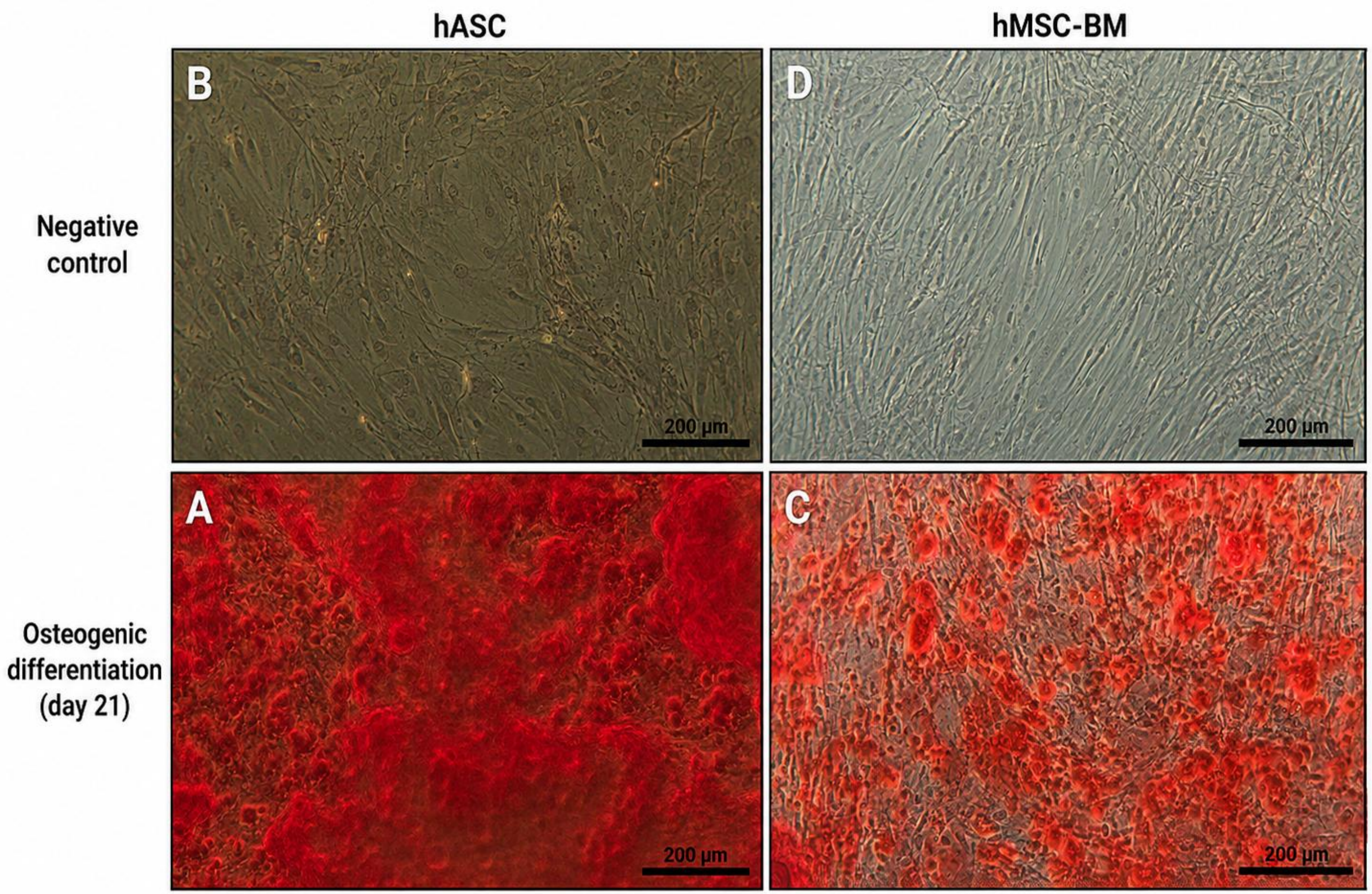

Supplementary Figure S1. Osteogenic differentiation capacity of hASC and hMSC-BM.
Representative brightfield micrographs of human adipose-derived stem cells (hASC) and human bone marrow-derived mesenchymal stem cells (hMSC-BM) after 21 days of culture in osteogenic differentiation medium (StemMACS™ OsteoDiff Medium, Miltenyi Biotec, Bergisch Gladbach, Germany) or corresponding growth-medium controls. Osteogenic differentiation was assessed by Alizarin Red S staining of calcium deposits (Merck KGaA, Darmstadt, Germany). Differentiation and staining were performed according to the manufacturers' protocols. hASC: (A) osteogenic differentiation and (B) negative control. hMSC-BM: (C) osteogenic differentiation and (D) negative control. Scale bars: 200 µm.
