# Supplementary material for: Parameter-resolved AC electrical stimulation links electrical system characteristics to early transcriptional responses in human stem cells
Source: Front Bioeng Biotechnol. 2026 Aug 25;14:1897534. doi: 10.3389/fbioe.2026.1897534 (PMC13550007; doi:10.3389/fbioe.2026.1897534)
Supplement: Supplementary file 2 [file Supplementaryfile1.pdf]

Supplementary Material 1

Electrical stimulation measurements and numerical electric-field simulation datasets

This supplementary material accompanies the article *“Parameter-resolved AC electrical stimulation links electrical system characteristics to early transcriptional responses in human stem cells”*.

The complete dataset underlying the electrical stimulation measurements and numerical electric-field simulations is approximately 1.9 GB and is therefore deposited in the publicly accessible Zenodo repository.

Permanent repository:

**Repository**	Zenodo
**DOI**	10.5281/zenodo.18466464
**Dataset file**	S1_ES_EField_DataAnalysis_Output.zip
**Approximate size**	1.9 GB

Contents

The repository contains the complete electrical stimulation measurement outputs and numerical electric-field simulation data supporting the quantitative electrical characterization and cell-layer-resolved field analyses reported in the article.

The Zenodo record provides permanent public access to the complete dataset and should be used to retrieve Supplementary Material 1.

Data citation: Lembcke, L. (2026). Supplement: Parameter-resolved AC electrical stimulation links electrical system characteristics to early transcriptional responses in human stem cells. Zenodo. DOI: 10.5281/zenodo.18466464
